# Complications of reversible cerebral vasoconstriction syndrome in relation to age

**DOI:** 10.1007/s00415-023-11708-z

**Published:** 2023-04-13

**Authors:** Kristin Sophie Lange, Gabrielle Tuloup, Claire Duflos, Claire Gobron, Cécilia Burcin, Lucas Corti, Caroline Roos, Anne Ducros, Jérôme Mawet

**Affiliations:** 1 Department of Neurology, CHU Montpellier, Gui de Chauliac Hospital, Montpellier, France; 2 Center for Stroke Research Berlin (CSB), Charité-Universitätsmedizin, Berlin, Germany; 3 Department of Neurology, Charité-Universitätsmedizin Berlin, Charitéplatz 1, 10117 Berlin, Germany; 4 Emergency Headache Center, Department of Neurology, Lariboisière Hospital, Assistance Publique des Hôpitaux de Paris, Paris, France; 5 Department of Neurology, CHU Caen-Normandie, Caen, France; 6 Clinical Research and Epidemiology Unit, Department of Public Health, CHU Montpellier, Montpellier University, Montpellier, France; 7 Department of Clinical Physiology, APHP, Lariboisière-St Louis Hospitals, DMU DREAM, 75010 Paris, France; 8 Charles Coulomb Laboratory, CNRS UMR5221, Montpellier University, Montpellier, France

**Keywords:** Cerebrovascular diseases, Stroke, Reversible cerebral vasoconstriction syndrome, Thunderclap headache, Intracerebral haemorrhage, Subarachnoid haemorrhage

## Abstract

**Introduction:**

Reversible cerebral vasoconstriction syndrome (RCVS) has a heterogenous clinical and radiological presentation. We investigated whether RCVS complications vary according to age.

**Patients and methods:**

In a pooled French cohort of 345 patients with RCVS, we assessed (1) rates of clinical and radiological complications, and (2) the functional outcome at 3 months according to age as a continuous variable, and in young patients aged ≤ 49 years versus those aged ≥ 50 years. The *Commission Nationale Informatique et Liberté* and the local ethics committee approved this study (registration number: 202100733).

**Results:**

The risk for any focal deficit and for any brain lesion were independently associated with increasing age (OR 1.4, 95% CI 1.1–1.8; *p* = 0.014, and OR 1.6, 95% CI 1.2–2.1; *p* < 0.001, respectively). Subtypes of brain lesions independently associated with increasing age were subarachnoid haemorrhage (OR 1.7, 95% CI 1.3–2.3; *p* < 0.001) and intracerebral haemorrhage (OR 1.5, 95% CI 1.1–2.2; *p* = 0.023). Frequency of cervical artery dissections peaked at age 30–39, and young age was independently associated with cervical artery dissections (OR 13.6, 95% CI 2.4–76.6; *p* = 0.003). Age had no impact on the functional outcome, with a modified Rankin scale score of 0–1 in > 96% of patients.

**Conclusion:**

Age seems to influence rates and types of complications of RCVS, with young age being associated with cervical artery dissections, and increasing age with haemorrhagic complications. If confirmed in larger prospective studies, recognition of age-specific patterns might help to guide clinical management and to identify complications in cases of RCVS and vice versa.

**Supplementary Information:**

The online version contains supplementary material available at 10.1007/s00415-023-11708-z.

## Introduction

Reversible cerebral vasoconstriction syndrome (RCVS) is characterised by severe acute headache associated with segmental cerebral vasoconstriction resolving within 3 months [1, 2]. It predominantly affects women in their forties or fifties, with an age range of 4 months to 85 years [3–5]. Clinical spectrum of RCVS is heterogeneous ranging from pure cephalalgic forms to complications including focal deficits, seizures, ischaemic stroke, intracranial haemorrhage, posterior reversible encephalopathy syndrome and cervical artery dissection [6–14]. Potential risk factors for such complications include female sex, postpartum, the absence of thunderclap headache at onset, and use of serotonergic antidepressants [8–10, 15].

Recently, analyses of the US Nationwide Readmission and Inpatient Databases identified advanced age as an independent risk factor for intracranial haemorrhage during RCVS, for higher length-of-stay in hospital, and for poor early outcome defined by discharge from hospital other-than-home [6, 16].

Studying risk factors for complications in RCVS is crucial in order to guide clinical management including the decision of ambulatory vs in-hospital care. We aimed to investigate whether clinico-radiological complications and prognosis of RCVS vary according to age.

## Materials and methods

### Study design and selection criteria

This is a pooled analysis of one prospective and two retrospective RCVS cohorts, which have been recruited at the French University Hospitals of Paris Lariboisière and Montpellier, as described before [9].

We included patients ≥ 15 years old with confirmed RCVS meeting the following criteria: (1) unusual, recent, severe headaches of sudden or progressive onset with or without focal neurologic deficit and/or seizures; (2) cerebral vasoconstriction with at least 2 narrowing on 2 different arteries, assessed by noninvasive brain vascular imaging investigations (CTA, contrast-enhanced MRA or Time-of-Flight (TOF)-MRA) or conventional cerebral angiography; and (3) disappearance of arterial abnormalities within the first 3 months.

At both hospitals, the performance of cerebral (CT, MRI) and cerebrovascular imaging investigations (CTA, MRA or conventional cerebral angiography) was left to the discretion of the physician in charge of the patient. All patients received vascular imaging at least once at onset to confirm vasoconstriction as defined above (criterion 2), and once at 3 months follow-up to confirm reversibility of vasoconstriction (criterion 3). Parenchymal imaging was repeated in case of clinical worsening. MR sequences at both hospitals included at least DWI, ADC, FLAIR and T2* or SWI, respectively.

At Lariboisière Hospital, all consecutive patients with proven RCVS were prospectively included from 2004 to 2011 (cohort A, *n* = 173). 70% of the patients were recruited from the emergency headache center [EHC], and 30% from the neurologic department including a stroke unit. Patient characteristics, treatment, and assessment of reversibility of vasoconstriction have been previously described [5]. All patients had a follow-up visit at 3 months in order to confirm clinical and radiological reversibility, and to assess the functional outcome. Further clinical follow-up visits were performed as previously described, with a mean follow-up period of 9.2 ± 3.3 years [17]. After the end of the prospective study at Lariboisière Hospital, patients were retrospectively included from 2012 to 2015, following the same inclusion criteria (cohort B, *n* = 132). 80% of the patients were recruited from the EHC, and 20% from the neurology department.

At the University Hospital of Montpellier, patients were retrospectively identified from the electronic database by using the International Classification of Diseases codes I67.84 and I67.9 from 2016 to 2019. For all identified cases (cohort C, *n* = 40), we analysed their medical records to ensure that they met the above-mentioned inclusion criteria. All identified patients had been admitted to the neurology department.

For both retrospective cohorts, clinical routine included a follow-up appointment at 3 months in order to clinically and radiologically confirm the diagnosis of RCVS according to the above-mentioned criteria, and to assess functional outcome.

Ethics approvals were obtained from the respective local institutional review boards. The database used in the study was approved by the *Commission Nationale Informatique et Liberté*. The study was conducted in accordance with the 1964 Declaration of Helsinki and its later amendments.

### Definitions of clinical characteristics

Migraine was diagnosed according to the International Classification of Headache Disorders [18]. A thunderclap headache (TCH) was defined as a headache reaching a maximum intensity above 7/10, on an 11-point scale from 0 to 10 in < 1 min.

We assessed the delay between onset, precisely the first episode of headache or neurological deficit, and the first neurological examination. RCVS was qualified as secondary in the presence of a potential precipitating factor and as idiopathic when none could be identified.

We collected data on the occurrence of clinical symptoms (i.e., transient focal neurological deficit < 24 h, persistent focal neurological deficit ≥ 24 h, and seizure) and of radiological lesions (i.e., ischaemic stroke [IS], intracerebral haemorrhage [ICH], subarachnoid haemorrhage [SAH], subdural haematoma [SDH], posterior reversible encephalopathy syndrome [PRES], and cervical artery dissections [CAD]), defined as complications of RCVS. Any clinical complication was defined as either any focal deficit and/or seizure. Any brain lesion was defined as ≥ 1 of the above-mentioned radiological lesions excluding CAD.

According to considerations for outcome definition in stroke trials [19], functional outcome was defined as good if the modified Rankin Scale (mRS) score at 3-month clinical follow-up was 0–1, and as unfavourable if the mRS score was ≥ 2.

### Statistical analysis

Continuous variables were presented as mean (± standard deviation, SD) if normally distributed and as medians [interquartile range, IQR] if not normally distributed. Categorical variables were presented as numbers (%). Categorical variables were compared between two groups by Pearson´s chi-squared test or the Fisher exact test if Chi-square’s conditions were not met. For continuous variables, the Mann–Whitney-*U*-test was applied for comparison of non-normally distributed data. The mRS was dichotomised as 0–1 vs 2–6.

The univariable relationship between age and clinical characteristics was assessed in comparing younger vs older patients (≤ 49 vs  > 50 years). We chose the cut-off of 50 years corresponding to the mean age of menopause, given the influence of female hormones in migraine and possibly in RCVS, and the high prevalence of migraine in patients with RCVS, decreasing after menopause [20–22].

The univariable relationship of age with clinical or radiological complications and with functional outcome was assessed graphically, using age groups (≤ 19, 20 to 29, 30 to 39, 40 to 49, 50 to 59, ≥ 60 years), and statistically using the cutoff of 50 years.

To determine independent associations of age with the occurrence of clinical or radiological complications and an unfavourable functional outcome (mRS ≥ 2), we performed a stepwise forward logistic regression. Results are presented as multivariable odds ratios and their 95% confidence intervals: OR [95% CI]. Depending on the shape of association with the outcome, age was used as a continuous variable with 10-year units, or as a categorical variable, with the a-priori cutoff of 50 years. To improve readability throughout the text, we label OR and *p* values OR^*C*^ and *p*^*C*^ when age was used as a continuous variable, and OR^*D*^ and *p*^*D*^ when age was used as a dichotomous variable, respectively.

Confounders known or supposed to be associated with age, with the occurrence of complications or unfavourable outcome in RCVS are: arterial hypertension, diabetes mellitus, hypercholesterolaemia, current smoking, cannabis use, cocaine use, blood pressure surge, thunderclap headache at onset, sex, migraine, postpartum, anxiety or depression, and serotonergic antidepressant use. All confounders independently associated with outcomes are presented in the results. Since age was our primary exposure, we included age in every model irrespective of a statistically significant association with the outcome.

Patients with missing values for one of the covariates were excluded from multivariable analysis. Results were considered significant at a two-sided alpha-level of 0.05. All statistical analyses were conducted using SPSS 25 (SPSS Inc., Chicago, USA).

## Results

### Clinical cohort characteristics (Table 1)

**Table 1:** Clinical characteristics of the overall cohort, comparison of RCVS patients aged ≤ 49 years and ≥ 50 years

Variable	All (*n* = 345)	Age ≤ 49 years (*n* = 240)	Age ≥ 50 years (*n* = 105)	*p* Value
Study center				0.763
APHP Paris Lariboisière	305 (88)	213 (89)	92 (88)	
Montpellier University Hospital	40 (12)	27 (11)	13 (12)	
Female sex	228 (66)	131 (55)	97 (92)	** < 0.001**
Comorbidities				
Arterial hypertension	37 (11)	15 (6)	22 (21)	** < 0.001**
Diabetes	9 (3)	1 (0.4)	8 (8)	** < 0.001**
Hypercholesterolaemia	31 (9)	8 (3)	23 (22)	** < 0.001**
Current smoking	110 (32)	94 (39)	16 (15)	** < 0.001**
Migraine	92 (27)	53 (22)	39 (37)	**0.004**
Anxiety or depression	73 (21)	38 (16)	35 (33)	** < 0.001**
Precipitating factors for RCVS				
None	126 (36)	80 (33)	46 (44)	0.063
Physical stress	97 (28)	71 (30)	26 (25)	0.359
Postpartum	29 (8)	29 (12)	0 (0)	** < 0.001**
Emotional stress	74 (21)	50 (21)	24 (23)	0.673
Vasoactive substance	148 (43)	109 (45)	39 (37)	0.153
Serotonergic antidepressant	37 (11)	18 (8)	19 (18)	**0.003**
Cannabis	62 (18)	61 (25)	1 (1)	** < 0.001**
Cocaine/crack	10 (3)	10 (4)	0 (0)	**0.034**
Triptans	14 (4)	7 (3)	7 (7)	0.136
Clinical manifestations				
Delay from onset to neurological exam, days	5 [3–9]	5 [3–9]	6 [3–9]	0.504
Hospitalisation	227 (66)	146 (61)	81 (77)	**0.003**
BP surge (*n* = 335)	77 (23)	50 (21)	27 (26)	0.255
Thunderclap headache at onset	281 (81)	192 (80)	89 (85)	0.295
Trigger for any headache episode (*n* = 344)				
Sexual activity	118 (34)	104 (43)	14 (13)	** < 0.001**
Other trigger^a^	42 (12)	19 (8)	23 (22)	** < 0.001**
Treatment				
Calcium antagonist (*n* = 338)	322 (93)	222 (93)	100 (95)	0.163

Bold values indicate *p* values < 0.05

Values are given as median [IQR] for continuous data and as *n* (%) for categorical data

*p* Value from Fisher exact test or Pearson Chi^2^ test for categorical variables, Mann–Whitney *U*-test for non-normally distributed continuous variables

*APHP* Assistance Publique-Hôpitaux de Paris; *BP surge* blood pressure surge > 160/90 mmHg

^a^Triggers other than typical triggers for RCVS listed in the ICHD3-criteria; other triggers include micturition, sudden movement

Comparison between the three pooled cohorts of RCVS patients has been presented and discussed before [9].

Mean age was 42 years (SD: 13, range 15–85). Five patients (1.4%) were younger than 18 years old, while patients ≥ 60 years represented 6.9% of the cohort. There was a female preponderance with two thirds of patients being women. A history of migraine was documented in 27% of patients.

Neurological examination in our institutions was performed with a median delay of 5 [IQR: 3–9] days after onset of symptoms. The most common triggers for headaches were sexual and physical activity. Two thirds of patients were hospitalised.

### Occurrence of neurological complications

Information on the occurrence of clinical complications was available for all patients. MRI scans were performed in 97.4% (336/345) of patients. The remaining nine patients had purely cephalalgic RCVS with recurrent TCHs, normal CT imaging, and a self-limited clinical course in the absence of immunosuppressant treatment. Focal deficits and seizures were documented in 16% and 4% of patients, respectively. 27% of patients had at least one type of brain lesion. The most frequent radiological lesions were SAH, followed by CAD and ICH.

### Clinical characteristics of patients aged ≤ 49 vs ≥ 50 years (Table 1)

Patients ≥ 50 years were significantly more often female (*p*^*D*^ < 0.001), reported more often cardiovascular comorbidities, and more frequently a history of migraine (*p*^*D*^ = 0.004). Exposure to cannabis and cocaine/crack was identified almost only in patients ≤ 49 years, while the use of serotonergic antidepressants was more common among patients ≥ 50 years (*p*^*D*^ < 0.001, *p*^*D*^ = 0.034; and *p*^*D*^ = 0.003, respectively).

While the percentage of patients presenting with TCH at onset was equal between both groups, older patients reported less often sexual triggers for headache episodes, and more often other triggers including micturition and sudden movement (*p*^*D*^ < 0.01, respectively). The frequency of physical effort, Valsalva maneuver, emotional stress and contact with water as headache triggers was similar between both groups. No difference was found concerning the delay from onset to the first neurological exam. Patients ≥ 50 years old were more frequently hospitalised (*p*^*D*^ = 0.003).

### Complications according to age

**Fig. 1:**
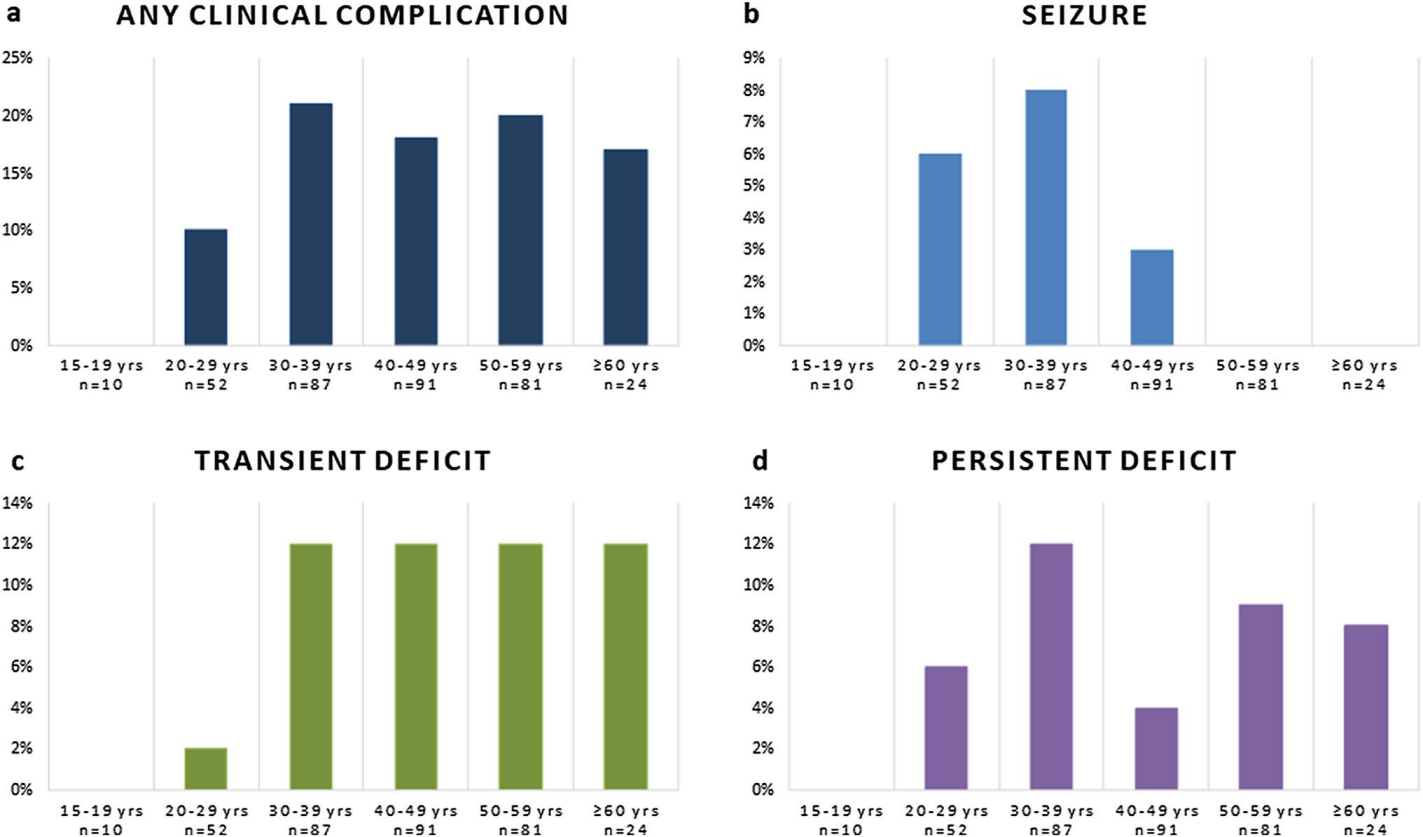
**a**–**d** Rates of clinical complications in RCVS according to age groups. Clinical complications are shown in percentages for each age group. *n* number; *yrs* years

**Fig. 2:**
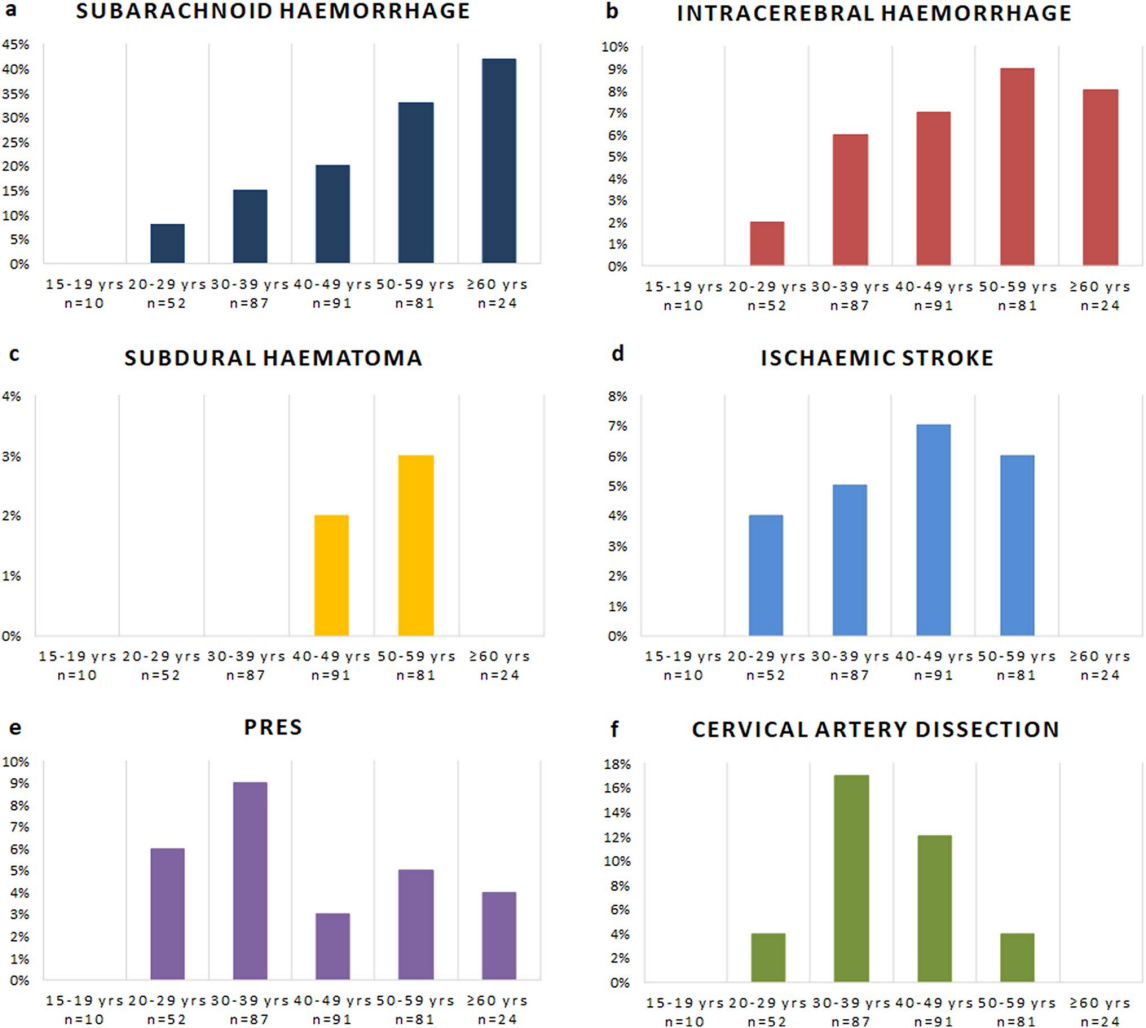
**a**–**f** Rates of radiological complications in RCVS according to age groups. Radiological complications are shown in percentages for each age group. *n* number; *PRES* posterior reversible encephalopathy syndrome; *yrs* years

Figures 1 and 2 display the frequency of clinical and radiological complications according to age groups, indicating percentages of patients in each age group affected by each type of complication. eTable 1 supplements the absolute numbers of complications. Regarding clinical complications, seizures occurred only in patients aged ≤ 49 years, while transient deficits seemed to increase up to the age of 39 years, and persistent deficits showed a bimodal distribution with two peaks at age 30–39 years and age ≥ 50 years.

The frequency of any brain lesion, SAH and ICH increased regularly across age groups. IS were almost equally distributed between age groups ≥ 20 years to ≤ 59 years, and PRES occurred at all ages ≥ 20 years. CAD peaked at age 30–49 years.

When comparing young patients ≤ 49 vs patients ≥ 50 years, older patients were significantly more often affected by any brain lesion (40% vs 21%, *p*^*D*^ < 0.001) and by SAH (35% vs 15%, *p*^*D*^ < 0.001), while younger patients presented significantly more often with seizures (5% vs 0%, *p*^*D*^ = 0.012) and CAD (12% vs 3%, *p*^*D*^ = 0.008, Table 2).

**Table 2:** Complications and outcome of the overall cohort, comparison of RCVS patients aged ≤ 49 years and ≥ 50 years

Variable	All (*n* = 345)	Age ≤ 49 years (*n* = 240)	Age ≥ 50 years (*n* = 105)	*p* Value
Clinical complications				
Any clinical complication^a^	59 (17)	39 (16)	20 (19)	0.525
Any focal deficit	55 (16)	35 (15)	20 (19)	0.297
Transient deficit < 24 h	35 (10)	22 (9)	13 (12)	0.363
Focal deficit $\ge$ 24 h	26 (7.5)	17 (7)	9 (9)	0.630
Seizure	13 (4)	13 (5)	0 (0)	**0.012**
Radiological complications				
Any brain lesion^b^, excluding cervical artery dissection	92 (27)	50 (21)	42 (40)	** < 0.001**
Ischaemic stroke	17 (5)	12 (5)	5 (5)	0.925
Intracerebral haemorrhage	21 (6)	12 (5)	9 (9)	0.202
Subdural haematoma	4 (1)	2 (1)	2 (2)	0.588
Subarachnoid haemorrhage	72 (21)	35 (15)	37 (35)	** < 0.001**
PRES	19 (5)	14 (6)	5 (5)	0.688
Cervical artery dissection	31 (9)	28 (12)	3 (3)	**0.008**
Functional outcome				
mRS at 3 months				0.291
0–1	333 (96.5)	230 (96)	103 (98)	
≥ 1	12 (3.5)	10 (4)	2 (2)	

Bold values indicate *p* values < 0.05

Values are n (%). P-value from Fisher exact test or Pearson Chi^2^ test. mRS, Modified Rankin Scale score; PRES, posterior reversible encephalopathy syndrome

^a^Including any focal neurological deficit < 24 h, ≥ 24 h, and seizures

^b^Including ischemic stroke and intracerebral haemorrhage, subdural haematoma, subarachnoid haemorrhage, and PRES

### Risk factors independently associated with the occurrence of neurological complications

Results of multivariable analysis are displayed in Table 3. No multivariable model was fitted for subdural hematoma because there were not enough events. After adjustment for potential confounders, increasing age was independently associated with the occurrence of any focal deficit (OR^*C*^ = 1.4 [1.1–1.8], *p*^*C*^ = 0.014). Other independent risk factors for any focal deficit were postpartum status and use of serotonergic antidepressants. Increasing age was independently associated with any brain lesion (OR^*C*^ = 1.6 [1.2–2.1], *p*^*C*^ < 0.001), SAH (OR^*C*^ = 1.7 [1.3–2.3], *p*^*C*^ < 0.001), and ICH (OR^*C*^ = 1.5 [1.1–2.2], *p*^*C*^ = 0.023). Other independent risk factors for any brain lesion were postpartum status, absence of TCH at onset, female sex, blood pressure surge and anxiety or depression, independently of the use of serotonergic antidepressants (data not shown). Age ≤ 49 years was an independent risk factor for CAD (OR^*D*^ = 13.6 [2.4–76.6], *p*^*D*^ = 0.003), alongside with hypercholesterolaemia, female sex, absence of TCH at onset and postpartum status. Increasing age was not associated with risk of ischaemic stroke or PRES (data not shown).

**Table 3:** Multivariable analysis on the association of clinical characteristics and occurrence of neurological complications in the pooled cohorts A–C (*n* = 335^a^)

Outcome	Variable	OR [95% CI]	*p* Value
Clinical complications			
Any focal deficit	Postpartum	7.5 [3.1–18.4]	< 0.001
	Serotonergic antidepressant	2.4 [1.0–5.4]	0.044
	Age (per 10 years increase)	**1.4 [1.1–1.8]**	**0.014**
Seizure	Postpartum	41.0 [9.3–181.7]	< 0.001
	BP surge	7.8 [1.9–32.7]	0.005
	Age ≤ 49 years	–	–
Radiological complications			
Subarachnoid haemorrhage	Female sex	3.3 [1.2–9.2]	0.023
	Absence of TCH at onset	2.9 [1.5–5.8]	0.002
	Postpartum	2.9 [1.1–7.9]	0.039
	Serotonergic antidepressant	2.9 [1.3–6.5]	0.012
	Migraine	1.9 [1.0–3.5]	0.049
	Age (per 10 years increase)	**1.7 [1.3–2.3]**	** < 0.001**
Intracerebral haemorrhage	Age (per 10 years increase)	**1.5 [1.1–2.2]**	**0.023**
Cervical artery dissection	Hypercholesterolaemia	17.9 [3.5–92.9]	0.001
	Age ≤ 49 years	**13.6 [2.4–76.6]**	**0.003**
	Female sex	8.1 [1.6–41.0]	0.011
	Absence of TCH at onset	5.7 [2.3–14.1]	< 0.001
	Postpartum	3.2 [1.1–9.0]	0.028

Bold values indicate *p* values < 0.05 for the variable age

OR [95% CI] and *p* value from logistic regression. OR of age could not be computed for seizures because no seizure occurred after 50 years

*BP surge* blood pressure surge > 160/90 mmHg; *TCH* thunderclap headache

^a^Patients with missing values for one of the covariates were excluded from multivariate analysis (*n* = 10)

### Functional outcome

Prognosis was good (mRS score 0–1) at 3 months for 96.5% of patients, without difference according to age groups. In univariable analysis, an unfavourable functional outcome (mRS ≥ 2) was more frequent in patients with SSRI/SNRI intake (13.5% vs 2.3%, *p* = 0.005), with IS (17.6% vs 2.7%, *p* = 0.017) and with ICH (33.3% vs 1.5%, *p* < 0.001). In multivariable analysis, the use of an SSRI/SNRI (OR = 5.5 [1.4–22.5], *p* = 0.017) and the occurrence of ICH (OR = 28.6 [7.6–107.2], *p* < 0.001) were independently associated with an unfavourable outcome.

## Discussion

The main finding of our study is that frequency and type of clinical and radiological complications in RCVS vary with age. While increasing age was independently associated with occurrence of a focal neurological deficit and haemorrhagic complications, young age was independently associated with cervical artery dissections.

We found an independent association between increasing age and the occurrence of a neurological deficit during the clinical course of RCVS. Other studies have reported transient or persistent deficits in 6–43% of RCVS cases, but none has investigated an association with age [7, 10]. Two studies examining risk factors for clinical worsening did not find an association between age and clinical worsening, while radiologic worsening was associated with advancing age [15, 23].

Seizures were associated with age ≤ 49 years in univariable analysis (*p*^*D*^ = 0.012), but this association could not be tested in a multivariable model because seizures occurred only in patients ≤ 49 years. Of note, the strongest independent predictor for seizures was postpartum, a condition affecting only younger women. Precisely, 10/13 patients presenting with seizures were in a postpartum context. Headache and seizures are also key features of eclampsia [24], and RCVS, PRES and eclampsia are presumed to belong to the same spectrum of pregnancy-related diseases involving endothelial dysfunction as one important pathophysiological mechanism [25, 26]. RCVS should be considered as an important differential or concomitant diagnosis for new-onset postpartum seizures in order to treat patients accordingly and to prevent complications [27].

With respect to brain lesions, subarachnoid haemorrhage was the most common complication in the entire cohort, followed by intracerebral haemorrhage. This finding is in line with the results of the above-cited observational study from the 2016 US Nationwide Readmission Database, reporting haemorrhagic complications in 43% of patients [16]. The authors identified female sex as well as middle to older age-group as independent risk factors for a combined outcome of ICH, SAH and SDH. Topcuoglu et al*.* compared haemorrhagic and nonhaemorrhagic RCVS, and found that female sex but not age was a predictor for intracerebral haemorrhage [28]. A former analysis of the first 89 patients from our cohort identified female sex and migraine as risk factors for haemorrhagic manifestations of RCVS [29]. In the present study, the number of cases allowed for a separate analysis of ICH and SAH, revealing an independent association of increasing age with ICH as well as SAH. Of note, increasing age was the only independent risk factor for ICH in our cohort. Regarding SAH, increasing age was the most significant risk factor. Other risk factors included the absence of TCH at onset, the use of serotonergic antidepressants, female sex, postpartum status and migraine. In line with previous studies [16, 28, 29], arterial hypertension and blood pressure surge were not independently associated with haemorrhages.

Concerning possible pathophysiological hypotheses, it has been shown in the general population that age-dependent changes in the mechanical and structural properties of the vascular wall lead to loss of arterial elasticity and reduced arterial compliance [30]. Hence, age is one of the most important risk factors for intracerebral haemorrhage [31]. In RCVS, arterial stiffness related to age might reduce vasospasm tolerance, thereby increasing the risk for rupture with age.

Population-based studies have associated age-dependent vascular stiffness and risk of cardiovascular events including IS in the elderly, and one study identified increased arterial stiffness as a predictor of delayed IS in patients with SAH [32, 33]. Among patients with RCVS, an analysis of the US Nationwide Readmissions Database 2016–2017 found a higher risk for IS in patients with hypertension, diabetes and tobacco use, but no association with age [34]. Likewise, in our cohort, age was not associated with IS, but the number of IS was low and larger studies are required.

CAD showed a non-linear association with age, peaking at 30–39 years. To date, the causal and chronological relation between CAD and RCVS remains unclear and has been the subject of previous publications [35–37]. In summary, RCVS might induce CAD by affecting vessel wall vasa vasorum, or by increasing pressure upstream of multiple intracranial stenosis in susceptible patients. Conversely, the occurrence of CAD might cause release of vasoactive substances which trigger RCVS. Regarding the general population, the large CADISP study reported a mean age of 41 years for vertebral artery dissections and 46 years for carotid artery dissections, respectively [38]. Established risk factors for CAD comprise genetic and environmental factors, including migraine, a condition that also predisposes to RCVS [36, 39]. Hypercholesterolaemia is not a risk factor for CAD and conversely, there is no evidence that atherosclerotic plaques are protective against CAD [40]. Surprisingly, we found an association between hypercholesterolaemia and CAD during RCVS. Since only five patients had CAD and hypercholesterolaemia, larger studies are warranted. The predominance of CAD in younger patients with RCVS is not surprising. Higher elasticity of vascular walls at younger age might predispose for the occurrence of a subintimal tear with consequent blood stream between the layers of intima and media, or for intramural rupture of vasa vasorum, with subsequent dissection of the media and adventitia, respectively. Nevertheless, this finding is of relevance for clinicians since it highlights the need for adequate and sometimes repeated imaging to detect both dissections and RCVS in patients with unusual headache.

These findings are of clinical relevance for several reasons. First, knowing the frequency and type of clinical and radiological complications of RCVS as a function of age could guide the choice of required brain and vascular imaging, and the decision of inpatient or outpatient treatment. Up to now, scores have been proposed to differentiate RCVS from PACNS, and to evaluate severity of vasoconstriction, but there is no score predicting complications [41, 42]. Second, our findings further underscore the necessity to consider RCVS in patients presenting with non-aneurysmal convexity SAH (cSAH) or atypical ICH in the absence of cerebral amyloid angiopathy (CAA). In a recent retrospective study on 70 patients with non-traumatic non-aneurysmal cSAH, CAA was the most common cause of unifocal cSAH, while RCVS was the commonest cause of multifocal SAH [43]. Another study of patients with cSAH found that RCVS was the most likely cause in patients ≤ 60 years whereas CAA was more likely in patients > 60 years [44]. In our cohort, 42% of patients ≥ 60 years presented with non-aneurysmal SAH, emphasizing that RCVS should also be considered in patients ≥ 60 years.

Aside from the distribution of complications, younger and older patients also differed with regards to sex, comorbidities, predisposing factors and triggers. As expected, cardiovascular comorbidities and use of serotonergic antidepressants were more frequent in older patients whereas use of illicit drugs, and sexual triggers for headache episodes were less frequent. Most importantly, the vast majority (> 90%) of the patients ≥ 50 years of age were women. In line with previous studies, these findings suggest a heterogenous spectrum of RCVS with the existence of subgroups of RCVS patients [7, 8, 10]. Young male patients often merely report use of illicit drugs, sexual triggers for headache and present a benign course of RCVS, young postpartum women seem to have higher prevalence of complications, and finally, older female patients seem to be characterised by a history of migraine, idiopathic RCVS or RCVS secondary to serotonergic antidepressants, and presence of haemorrhagic lesions on imaging. Larger studies with cluster analyses, such as conducted in transient global amnesia [45], a condition sharing numerous features with RCVS, are necessary to statistically confirm the existence of such subgroups.

The overall clinical outcome at 3 months of follow-up in our cohort was very good with an mRS score 0–1 in 96.5% of patients. Contrarily, an analysis of the US Nationwide Inpatient Database including 2020 patients with RCVS found a poor early outcome of discharge to another facility after hospitalisation or in-hospital death in one third of patients [6]. This discrepancy might be explained mainly by different recruitment patterns. While the US study described a population of hospitalised patients, two third of our patients were recruited from an emergency headache center, and one third of our patients were treated on an outpatient basis [9]. While the emergency headache center is easily accessible for any patient with a headache, capacities of our neurovascular units are restricted, thus limiting the transfer of severely affected patients from other hospitals or departments. This might have contributed to a selection bias for milder forms in our cohort with underestimation of the outcome severity. On the other hand, pure and mild cephalagic forms of RCVS might also be underrepresented, since those patients might not consult or might not return for 3 months follow-up, rendering impossible the definite diagnosis of RCVS. Overall, recruitment from two neurological wards and one emergency headache center might have balanced selection bias for milder cephalalgic vs more severe forms, respectively.

In our cohort, predictors for an unfavourable functional outcome included the use of serotonergic antidepressants prior to RCVS onset, and the occurrence of IS or ICH during RCVS. Only the use of serotonergic antidepressants and the occurrence of ICH were independent predictors. Prior studies have identified IS as the most consistent predictor [6, 15, 23, 34], and ICH as another independent predictor [6] for a poor functional outcome, underlining the importance of careful monitoring and rehabilitative measures in patients with these types of lesions. Concerning serotonergic antidepressants, prior studies have found an association with clinical and angiographic worsening but not poor outcome (defined as mRS > 3) [15]. Taken together, these findings suggest that patients with use of serotonergic antidepressants might benefit from in-hospital care allowing for close monitoring.

Contrarily to two prior studies [6, 46], we did not find an association of advanced age and an unfavourable outcome. In our cohort, patients aged > 50 years were significantly more often hospitalised. The in-patient stay might have allowed for a prevention of clinical worsening, e.g. by the means of blood pressure management and physical rest.

The main limitation of our study consists in the retrospective data acquisition for cohorts B and C. Further, although we describe the largest European cohort to date, the numbers for some subtypes of complications remain small. This possibly contributes to the inconsistency of findings between the different cohorts published to date, and future larger multi-centric studies could be suited to confirm or refute clinical and radiological predictors of complications and of an unfavourable functional outcome. Moreover, since we included only adult patients aged ≥ 15 years from two French hospitals, our findings may not be applicable to other populations and to pediatric patients. Finally, we have not compared the localisation, degree and number of vasospasms, possibly varying according to age and influencing the risk for complications.

## Conclusion

Among adult patients with RCVS in France, rates and types of complications vary according to age. While young age was associated with CAD, increasing age predisposed for haemorrhagic complications. Larger multi-center studies are necessary to confirm possible age-specific presentations of RCVS and should consider age when developing prognostic scores helping to guide clinical decision-making.

## Supplementary Information

Below is the link to the electronic supplementary material.

Supplementary file1 (DOCX 18 KB)

## Data Availability

The data that support the findings of this study are available from the corresponding author, upon reasonable request.

## Abbreviations

ADC: Apparent diffusion coefficient
CAA: Cerebral amyloid angiopathy
CAD: Cervical artery dissection
CI: Confidence interval
CT: Computer tomography
CTA: Computed tomography angiography
DWI: Diffusion-weighted imaging
EHC: Emergency headache center
FLAIR: Fluid-attenuated inversion recovery
ICH: Intracerebral haemorrhage
IS: Ischaemic stroke
IQR: Interquartile range
MRI: Magnetic resonance tomography
MRA: Magnetic resonance angiography
mRS: Modified Rankin scale
OR: Odds ratio
OR^*C*^: Odds ratio, age used as a continuous variable
OR^*D*^: Odds ratio, age used as a dichotomous variable
*p*^*C*^: *p* Value, age used as a continuous variable
*p*^*D*^: *p* Value, age used as a dichotomous variable
PRES: Posterior reversible encephalopathy syndrome
RCVS: Reversible cerebral vasoconstriction syndrome
SAH: Subarachnoid haemorrhage
cSAH: Convexity subarachnoid hemorrhage
SD: Standard deviation
SDH: Subdural haematoma
SSRI: Selective serotonin reuptake inhibitors
SNRI: Serotonin and noradrenaline reuptake inhibitors
SWI: Susceptibility-weighted imaging
TCH: Thunderclap headache

## Declarations

### Guarantor

KSL.
