# Supplementary material for: Complications of reversible cerebral vasoconstriction syndrome in relation to age
Source: J Neurol. 2023 Apr 13;270(7):3584–94. doi: 10.1007/s00415-023-11708-z (PMC10267248; doi:10.1007/s00415-023-11708-z)
Supplement: Supplementary file 1 — Supplementary file1 (DOCX 18 KB) [file 415_2023_11708_MOESM1_ESM.docx]

# Lange et al. Complications of reversible cerebral vasoconstriction syndrome in relation to age

# Table 1. Clinical and radiological complications according to age groups.

| Age groups | 15-19 years  n=10 | 20-29 years  n=52 | 30-39 years  n=87 | 40-49 years  n=91 | 50-59 years  n=81 | ≥60 years  n=24 |  |
| --- | --- | --- | --- | --- | --- | --- | --- |
| Outcome |  |  |  |  |  |  |  |
| Clinical complications | | | | | | |  |
| Any clinical complication† | 0 (0) | 5 (10) | 18 (21) | 16 (18) | 16 (20) | 4 (17) |  |
| Any focal deficit | 0 (0) | 3 (6) | 18 (21) | 14 (15) | 16 (20) | 4 (17) |  |
| Transient deficit | 0 (0) | 1 (2) | 10 (12) | 11 (12) | 10 (12) | 3 (12) |  |
| Persistent deficit ≥24 hours | 0 (0) | 3 (6) | 10 (12) | 4 (4) | 7 (9) | 2 (8) |  |
| Seizure | 0 (0) | 3 (6) | 7 (8) | 3 (3) | 0 (0) | 0 (0) |  |
| Radiological complications | | | | | | |  |
| Any brain lesion‡ | 0 (0) | 6 (12) | 21 (24) | 23 (25) | 31 (38) | 11 (46) |  |
| Ischemic stroke | 0 (0) | 2 (4) | 4 (5) | 6 (7) | 5 (6) | 0 (0) | |
| Intracerebral hemorrhage | 0 (0) | 1 (2) | 5 (6) | 6 (7) | 7 (9) | 2 (8) |  |
| Subdural hematoma | 0 (0) | 0 (0) | 0 (0) | 2 (2) | 2 (3) | 0 (0) |  |
| Subarachnoid hemorrhage | 0 (0) | 4 (8) | 13 (15) | 18 (20) | 27 (33) | 10 (42) |  |
| PRES | 0 (0) | 3 (6) | 8 (9) | 3 (3) | 4 (5) | 1 (4) |  |
| Cervical artery dissection | 0 (0) | 2 (4) | 15 (17) | 11 (12) | 3 (4) | 0 (0) |  |

Values are given as n (%). PRES, posterior reversible encephalopathy syndrome. †Including any neurological deficit <24 hours, ≥24 hours, and seizures.

‡Including ischemic stroke, intracerebral hemorrhage, subdural hematoma, subarachnoid hemorrhage, posterior reversible encephalopathy syndrome, excluding

cervical artery dissections.
